# Low vs. High Inspiratory Oxygen Fraction During Mechanical Ventilation in Obese Patients: Impact on Postoperative Pulmonary Outcomes

**DOI:** 10.1155/anrp/5336172

**Published:** 2025-10-06

**Authors:** Xuelong Zhou, Jia Liu, Jingming Zhu, Xiuhong Jiang, Qi Zou

**Affiliations:** ^1^Department of Anesthesiology, Women's Hospital, School of Medicine, Zhejiang University, Hangzhou 310006, China; ^2^Department of Anesthesiology and Perioperative Medicine, The First Affiliated Hospital With Nanjing Medical University, Nanjing 210029, Jiangsu, China

**Keywords:** inspiratory oxygen fraction, mechanical ventilation, obese patients, postoperative pulmonary outcomes

## Abstract

**Background:**

Obese patients undergoing general anesthesia are at heightened risk for postoperative pulmonary complications (PPCs) due to impaired respiratory mechanics. While lung-protective ventilation strategies are widely adopted, the role of intraoperative inspiratory oxygen fraction (FiO_2_) in this population remains controversial.

**Methods:**

We conducted a prospective, randomized controlled trial comparing low (FiO_2_ 40%) versus high (FiO_2_ 80%) intraoperative oxygen concentration in 113 obese patients undergoing elective laparoscopic bariatric surgery. All patients received standardized lung-protective ventilation. The primary outcome was the incidence and severity of PPCs within 5 postoperative days. Secondary outcomes included hospital length of stay, mortality, postoperative nausea and vomiting (PONV), and surgical site infection (SSI).

**Results:**

The incidence of PPCs was 70.7% in the high FiO_2_ group and 55.5% in the low FiO_2_ group (*p*=0.08). Grade ≥ 3 PPCs occurred only in the high FiO_2_ group (3.4%). The average PPC severity score was lower in the low FiO_2_ group (0.9 ± 0.9 vs. 1.2 ± 0.9, *p*=0.08). No differences were observed in hospital stay, mortality, or SSI. PONV was more frequent in the low FiO_2_ group (43.6% vs. 27.6%, *p*=0.08).

**Conclusion:**

Although the difference was not statistically significant, the observed lower incidence of PPCs in the low FiO_2_ group may indicate a potential clinical effect. Larger, multicenter trials are warranted to confirm these results and optimize oxygen management in obese surgical patients.

**Trial Registration:** Chinese Clinical Trial Registry: ChiCTR2300076656

## 1. Introduction

Obesity is increasingly prevalent worldwide and presents a series of unique challenges in the perioperative period. Among these, the risk of postoperative pulmonary complications (PPCs) is significantly elevated in obese patients undergoing general anesthesia with mechanical ventilation. Due to altered respiratory mechanics, decreased functional residual capacity, increased airway resistance, and a predisposition to atelectasis, obese patients are particularly vulnerable to perioperative respiratory impairment [1]. The development of PPCs such as atelectasis, pneumonia, and hypoxemia not only prolongs hospital stay but also contributes to increased morbidity and healthcare costs [2].

To mitigate the risk of PPCs, lung-protective ventilation strategies—typically involving low tidal volume, optimal positive end-expiratory pressure (PEEP), and recruitment maneuvers—have become a cornerstone in intraoperative respiratory management [3]. However, in addition to these parameters, the inspiratory oxygen fraction (FiO_2_) used during mechanical ventilation may independently influence postoperative outcomes. Traditionally, high FiO_2_ levels (60%–100%) have been routinely administered during general anesthesia to prevent hypoxemia [4]. Yet, mounting evidence has raised concerns about the potential adverse effects of hyperoxia, including the promotion of resorption atelectasis, oxidative stress, and impaired mucociliary clearance, all of which may contribute to pulmonary complications [5].

Several studies in nonobese surgical populations have suggested that high intraoperative FiO_2_ may increase the risk of PPCs [6, 7], while others have reported no significant difference in outcomes between high and low FiO_2_ groups [8, 9]. These inconsistent findings reflect the heterogeneity in patient populations, surgical procedures, and ventilation strategies employed. Importantly, the impact of intraoperative FiO_2_ on postoperative outcomes in obese patients—a group with unique physiological and anatomical characteristics—remains largely unexplored. Whether the use of low FiO_2_ during mechanical ventilation can reduce the incidence of PPCs in this high-risk population is still unclear.

Therefore, to address this gap in evidence, we designed a prospective randomized controlled trial to compare the effects of low (e.g., 40%) versus high (e.g., 80%) intraoperative FiO_2_ in obese patients undergoing elective surgery, under a standardized lung-protective ventilation protocol. We hypothesized that using a lower FiO_2_ concentration would result in a reduced incidence and severity of PPCs in obese individuals.

## 2. Methods

### 2.1. Study Design

This study was designed as a prospective, randomized, controlled, parallel-group clinical trial. The study protocol was reviewed and approved by the Ethics Committee of Jiangsu Provincial People's Hospital on 11 January 2023 (Approval No. 2022-SR-737). The trial adhered to the principles of the Declaration of Helsinki and Good Clinical Practice (GCP) guidelines. All participants provided written informed consent prior to enrollment.

### 2.2. Study Population

Patients enrolled in this study were adult individuals scheduled to undergo elective bariatric surgery at Jiangsu Provincial People's Hospital between October 2023 and March 2024. All patients were screened preoperatively for eligibility according to predefined inclusion and exclusion criteria. Inclusion criteria were as follows: age between 18 and 65 years; body mass index (BMI) ≥ 30 kg/m^2^; American Society of Anesthesiologists (ASA) physical status classification II-III; scheduled for elective laparoscopic bariatric surgery under general anesthesia; provided written informed consent. Exclusion criteria included pre-existing pulmonary disease (e.g., chronic obstructive pulmonary disease, restrictive lung disease, or uncontrolled asthma); requirement for emergency surgery; preoperative hypoxemia (SpO_2_ < 90% on room air); history of heart failure (New York Heart Association Class III–IV) or recent myocardial infarction (< 6 months); pregnancy or lactation; known hypersensitivity to anesthetic agents used in the study protocol; participation in another interventional clinical trial within the past 30 days. Eligible patients were approached during the preoperative anesthesia consultation and enrolled after obtaining written informed consent. Baseline demographic and clinical data were collected prior to randomization.

### 2.3. Randomization and Blinding

After confirming eligibility and obtaining written informed consent, enrolled patients were randomly assigned in a 1:1 ratio to receive either high FiO_2_ (80%) or low FiO_2_ (40%) during intraoperative mechanical ventilation. Randomization was performed using a computer-generated random sequence, which was created by an independent statistician who was not involved in patient recruitment or data analysis. Allocation concealment was ensured through the use of sequentially numbered, opaque, sealed envelopes that were opened only after induction of anesthesia. Due to the nature of the intervention, the attending anesthesiologists could not be blinded to group assignment. However, all postoperative outcome assessors, data collectors, and statisticians remained blinded to the allocation throughout the study period to minimize assessment and analytical bias. To maintain blinding, the FiO_2_ settings were not documented in the standard anesthesia records accessible to outcome assessors, and intraoperative personnel were instructed not to disclose group assignments during postoperative evaluation.

### 2.4. Interventions and Anesthesia

Upon arrival in the operating room, standard monitoring was applied to all patients, including electrocardiography, noninvasive blood pressure, pulse oximetry, and end-tidal carbon dioxide (EtCO_2_). A peripheral intravenous line was established, and supplemental oxygen (FiO_2_ 100%) was administered via face mask for 3–5 min prior to induction. General anesthesia was induced using intravenous propofol (2–2.5 mg/kg), sufentanil (0.3–0.5 μg/kg), and rocuronium (0.6–0.9 mg/kg) to facilitate endotracheal intubation. Following confirmation of correct endotracheal tube placement, mechanical ventilation was initiated with a volume-controlled mode on an anesthesia machine (Dräger Primus or equivalent). Anesthesia was maintained with sevoflurane in a mixture of oxygen and air, and intermittent boluses of rocuronium and sufentanil were administered as needed to maintain muscle relaxation and hemodynamic stability. Core temperature was monitored continuously and maintained above 36.0°C using a forced-air warming device. Patients were ventilated using a standardized lung-protective ventilation strategy throughout the surgery. This included a tidal volume (VT) of 6–8 mL/kg of predicted body weight (PBW), a respiratory rate adjusted to maintain EtCO_2_ between 35 and 45 mmHg, and a PEEP of 8–10 cm H_2_O. Recruitment maneuvers were performed after intubation and surgery. Each recruitment maneuver consisted of applying a sustained inflation pressure of 30–40 cmH_2_O for 10–15 s. Patients were randomized to receive either a high (80%) or low (40%) FiO_2_ throughout the intraoperative period. The designated FiO_2_ was set immediately after the start of mechanical ventilation and maintained until the end of surgery. The balance of the inspired gas mixture was composed of medical air to achieve the target FiO_2_. FiO_2_ levels were monitored and continuously recorded by the anesthesia machine. Intraoperative fluid administration, vasopressor use, and other hemodynamic management strategies were guided by institutional protocols and adjusted by the attending anesthesiologist based on clinical judgment. No additional alveolar recruitment or FiO_2_ titration was performed unless clinically indicated (e.g., desaturation or hemodynamic instability). At the end of surgery, neuromuscular blockade was reversed with sugammadex, and patients were extubated when fully awake and meeting standard extubation criteria. Postoperative care, including oxygen supplementation and monitoring, was provided in the postanesthesia care unit (PACU) according to institutional standards.

## 3. Outcomes

The primary outcome of this study was the incidence and severity of PPCs within the first 5 days after surgery. PPCs were assessed daily by a blinded investigator using a standardized 6-point ordinal grading scale proposed by Costa et al. Grade 0 on the scale indicates the absence of symptoms or signs of PPCs, while Grades 1 to 4 reflect progressively more severe complications, and Grade 5 denotes death prior to hospital discharge. Each patient's PPC severity was recorded daily, and the worst grade observed during the 5-day postoperative period was used for analysis.

Secondary outcomes included the following: length of hospital stay, measured in days from the date of surgery to discharge; in-hospital mortality, defined as death from any cause during the index hospitalization; 30-day postoperative mortality, assessed via hospital records and telephone follow-up if necessary; incidence of postoperative nausea and vomiting (PONV), recorded during the first 24 h after surgery and treated according to institutional protocols; incidence of surgical site infection (SSI), defined according to CDC criteria, and diagnosed during hospitalization.

All outcome assessments were performed by investigators blinded to group allocation to reduce bias. Standardized definitions and data collection procedures were used to ensure consistency across evaluators.

### 3.1. Sample Size Calculation and Statistical Analysis

#### 3.1.1. Sample Size Calculation

The sample size was calculated based on the primary outcome: the incidence of PPCs within 5 days after surgery. Based on our pilot study, we assumed that the incidence of PPCs in the high FiO_2_ group would be approximately 75% and that a clinically meaningful reduction to 50% could be achieved in the low FiO_2_ group. Using a two-sided chi-square test with a significance level (α) of 0.05 and a power (1 − β) of 80%, a minimum of 55 patients per group was required. To account for potential dropouts and protocol violations, the final target sample size was set at 60 patients per group, totaling 120 patients.

#### 3.1.2. Statistical Analysis

All statistical analyses were performed using SPSS software (Version 18.0, IBM Corp., Armonk, NY, USA). Categorical variables, including the incidence and severity of PPCs, PONV, SSI, and mortality, were expressed as counts and percentages, and compared between groups using the chi-square test or Fisher's exact test, as appropriate. Continuous variables, such as length of hospital stay, were presented as mean ± standard deviation (SD) or median (interquartile range), depending on normality assessed by the Shapiro–Wilk test. Comparisons between groups were made using the independent samples *t*-test or Mann–Whitney U test. *p* < 0.05 was considered statistically significant.

## 4. Results

### 4.1. Patient Characteristics

A total of 136 patients scheduled for elective bariatric surgery were screened for eligibility. Among them, 16 patients were excluded due to not meeting inclusion criteria (*n* = 9) and refusal to participate (*n* = 7). The remaining 120 patients were randomly assigned to the low FiO_2_ group (*n* = 60) or the high FiO_2_ group (*n* = 60).

**Figure 1:**
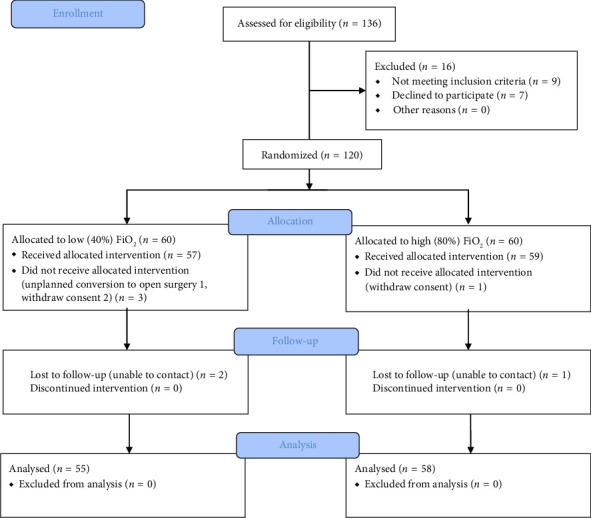
Flow diagram of the trial. FiO_2_, inspiratory oxygen fraction.

During the study, 5 patients in the low FiO_2_ group and 2 patients in the high FiO_2_ group were excluded from the final analysis due to unplanned conversion to open surgery (1), withdraw consent before anesthesia (3), and loss of follow-up data (3). As a result, a total of 113 patients (55 in the low FiO_2_ group and 58 in the high FiO_2_ group) were included in the final intention-to-treat analysis (Figure 1).

Baseline demographic and intraoperative characteristics were well balanced between the two groups and are summarized in Tables 1 and 2. There were no statistically significant differences between the groups in terms of age, sex, BMI, ASA physical status classification, comorbidities (e.g., hypertension and diabetes), smoking history, or surgery data. Intraoperative mechanical ventilation parameters, including tidal volume, PEEP, peak and plateau airway pressures, as well as gas exchange indices, are presented in Table S1. No statistically significant differences were observed between the two groups.

### 4.2. Primary Outcome

**Figure 2:**
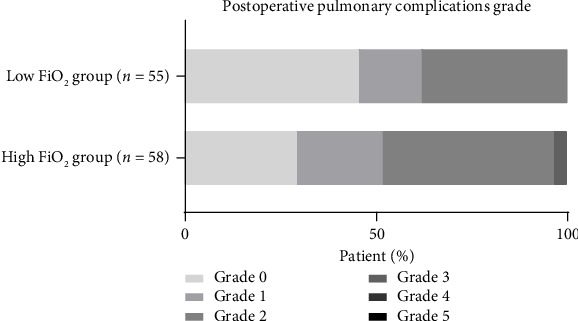
Severity grade of postoperative pulmonary complications after surgery. The severity grade of pulmonary complications scored by a scale ranging from 0 to 5. Grade 0 represents no symptoms or signals of PPCs; Grades 1 through 4 represent successively the worse forms of complications; and Grade 5 represents death before hospital discharge.

A total of 41 patients (70.7%) in the high FiO_2_ group developed PPCs of any grade, compared with 30 patients (55.5%) in the low FiO_2_ group, and the difference was not statistically significant (*p*=0.08). When stratified by severity, Grade 0 (no PPCs) occurred in 45.5% of the low FiO_2_ group compared to 29.3% in the high FiO_2_ group (*p*=0.08). Grades 1 and 2 were comparable between groups. Two patients (3.4%) in the high FiO_2_ group developed Grade 3 PPCs, while none in the low FiO_2_ group experienced severe complications (Grade ≥ 3) (*p*=0.50) (Table 3, Figure 2).

The average PPC severity score was 0.9 ± 0.9 in the low FiO_2_ group and 1.2 ± 0.9 in the high FiO_2_ group, with no statistically significant difference between the two groups (*p*=0.08).

### 4.3. Secondary Outcomes

As shown in Table 3, there was no significant difference in the length of hospital stay between groups, with both the low and high FiO_2_ groups having a mean duration of 2.1 ± 0.3 days (*p*=0.99). No in-hospital or 30 day mortality occurred in either group.

The incidence of PONV was higher in the low FiO_2_ group (43.6%) compared to the high FiO_2_ group (27.6%), though this difference did not reach statistical significance (*p*=0.08). Interestingly, despite prior concerns that high FiO_2_ might reduce PONV through reduced gut ischemia or less emetogenic stimuli, our data suggest a nonsignificant difference favoring less PONV in the high FiO_2_ group, warranting further investigation.

SSI occurred in only one patient (1.7%) in the high FiO_2_ group, and none in the low FiO_2_ group, with no significant difference between groups (*p*=0.99).

Overall, the secondary outcomes demonstrated no statistically significant differences.

## 5. Discussion

Intraoperative oxygen therapy, particularly the choice of FiO_2_, remains a critical and debated component of perioperative respiratory management. Obese patients are particularly susceptible to pulmonary complications due to altered respiratory mechanics, including decreased functional residual capacity, increased airway resistance, and a propensity for atelectasis [1]. Therefore, identifying optimal ventilatory strategies in this population is of paramount importance.

Our study compared the effects of low (40%) versus high (80%) intraoperative FiO_2_ on postoperative pulmonary and systemic outcomes in obese patients undergoing laparoscopic bariatric surgery under standardized lung-protective ventilation. Although our findings did not achieve statistical significance at the conventional threshold, the observed differences favored the low FiO_2_ group, particularly in terms of reduced incidence and severity of PPCs. Specifically, the overall incidence of PPCs was numerically lower in the low FiO_2_ group (55.5%) compared to the high FiO_2_ group (70.7%, *p*=0.08). Grade 0 PPCs (i.e., absence of complications) were more frequent in the low FiO_2_ group (45.5% vs. 29.3%), and no patients in the low FiO_2_ group developed severe PPCs (Grade ≥ 3), whereas two patients in the high FiO_2_ group did. While none of these differences reached statistical significance individually, the consistency of direction across PPC incidence, severity grading, and composite severity scores (0.9 vs. 1.2, *p*=0.08) suggests a clinically meaningful benefit of lower FiO_2_ that merits attention.

These observations are consistent with accumulating evidence that high intraoperative FiO_2_ can promote resorption atelectasis, impair mucociliary clearance, and exacerbate oxidative injury, especially in at-risk populations such as the obese [5, 10, 11]. Several studies and meta-analyses have reported a dose-dependent relationship between higher FiO_2_ and increased risk of PPCs, particularly when FiO_2_ exceeds 60% [6, 11, 12]. By contrast, lower FiO_2_ strategies (e.g., 30%–40%) combined with lung-protective ventilation have been associated with lower rates of PPCs [3, 8]. Our findings lend further support to this emerging paradigm, even if statistical power was insufficient to confirm significance.

In terms of secondary outcomes, no significant differences were noted in hospital stay, in-hospital mortality, or 30 day mortality. Notably, the incidence of PONV was numerically higher in the low FiO_2_ group (43.6% vs. 27.6%), though the difference was not statistically significant (*p*=0.08). This finding is somewhat contrary to prior literature suggesting that high FiO_2_ may increase the risk of PONV due to oxidative stress or gastrointestinal mucosal ischemia [11]. PONV is influenced by multiple factors, including anesthetic agents, opioid consumption, and individual patient susceptibility. Therefore, the observed difference toward less PONV in the high FiO_2_ group should be interpreted with caution, as this study was not designed or powered to assess PONV and did not control for all potential confounders. SSI was rare in both groups, with no meaningful differences observed.

Several factors may explain the lack of statistical significance despite the observed differences favoring the low FiO_2_ group. First, although the overall incidence of PPCs was high—particularly in the high FiO_2_ group—the incidence of severe PPCs (Grade ≥ 3) was low across both groups, which may have limited the study's power to detect statistically significant differences in more clinically impactful events. Second, while the sample size was calculated to detect a moderate effect size, it may still have been insufficient to identify smaller yet clinically meaningful differences, particularly given the variability inherent in PPC assessments. Third, all patients received a standardized lung-protective ventilation strategy with recruitment maneuvers, which may have mitigated the deleterious effects of high FiO_2_ and reduced between-group variability. Finally, although PPCs were evaluated using a validated ordinal grading scale, subclinical impairments—such as transient atelectasis or mild inflammatory changes—may have gone undetected, potentially underestimating the true impact of oxygen concentration on postoperative lung function.

In this study, a near-significant difference in the use of colloid solutions was observed between the two groups, despite comparable total intraoperative fluid volumes and no statistically significant differences in intraoperative hemodynamic events. This imbalance is unlikely to be related to the assigned FiO_2_ strategy and is more plausibly explained by variations in individual anesthesiologists' fluid management preferences. Additionally, small-sample random variation may have led to a disproportionate distribution of cases with slightly higher intraoperative fluid requirements in one group.

This study has several limitations. It was conducted at a single center with a relatively homogeneous population, limiting generalizability. The open-label nature of intraoperative management (due to the impossibility of blinding FiO_2_ settings for anesthesiologists) may introduce subtle performance bias, though outcome assessors remained blinded. Additionally, we only evaluated short-term outcomes up to 5 days postoperatively; longer-term respiratory sequelae were not captured.

In conclusion, although the differences between FiO_2_ groups did not achieve statistical significance, our study observed lower rates of pulmonary complications with low FiO_2_ (40%) under lung-protective ventilation in obese patients undergoing laparoscopic surgery. These findings suggest a potential clinical benefit and justify further investigation through larger, multicenter trials with extended follow-up. Until such data are available, cautious use of intraoperative oxygen, avoiding unnecessarily high FiO_2_, may be advisable in this vulnerable population.

## Figures and Tables

**Table 1:** Preoperative baseline characteristics of patients.

Variables	Low FiO_2_ group (*n* = 55)	High FiO_2_ group (*n* = 58)	*p* value
Age (years)	34.8 ± 8.5	34.5 ± 9.5	0.86
Sex (male)	20 (36.4)	22 (37.9)	0.99
Height (cm)	170.6 ± 7.8	169.0 ± 8.6	0.30
Weight (kg)	115.8 ± 22.9	109.3 ± 26.0	0.16
BMI (kg/m^2^)	39.5 ± 5.6	37.9 ± 7.3	0.20
ASA physical status			
II	30 (54.5)	35 (60.3)	0.57
III	25 (45.5)	23 (39.7)	0.57
ARISCAT score			
Intermediate risk	48 (87.3)	50 (86.2)	0.99
High risk	7 (12.7)	8 (13.8)	0.99
Comorbidity			
Smoking history	12 (21.8)	11 (19.0)	0.82
OSAS	13 (23.6)	14 (24.1)	0.99
Hypertension	11 (20.0)	11 (19.0)	0.99
Diabetes	13 (23.6)	16 (27.6)	0.67
Laboratory measurements			
Oxygenation index	406.9 ± 57.0	420.9 ± 86.1	0.31
WBC (× 10^9^/L)	7.7 ± 1.9	7.4 ± 1.7	0.38
Hemoglobin (g/L)	141.6 ± 19.6	142.2 ± 13.3	0.85
Creatinine (umol/L)	61.0 ± 12.9	56.1 ± 16.7	0.08
Albumin (g/L)	43.7 ± 2.4	44.6 ± 3.8	0.14

*Note:* ARISCAT, the Assess Respiratory Risk in Surgical Patients in Catalonia.

Abbreviations: ASA, American Society of Anesthesiologists; BMI, body mass index; OSAS, obstructive sleep apnea syndrome; WBC, white blood cell.

**Table 2:** Intraoperative characteristics of patients.

	Low FiO_2_ group (*n* = 55)	High FiO_2_ group (*n* = 58)	*p* value
Type of surgery			
SG	26 (47.3)	28 (48.3)	0.99
SG + JIB	25 (45.4)	24 (41.4)	0.71
Other	4 (7.3)	6 (10.3)	0.74
Type of anesthesia			
CIVIA	55 (100.0)	58 (100.0)	0.99
Duration of surgery (min)	79.1 ± 23.5	76.0 ± 23.9	0.49
Duration of anesthesia (min)	89.2 ± 23.5	88.9 ± 24.8	0.95
Dosage of analgesic			
Remifentanil (ug)	487.2 ± 181.9	457.5 ± 164.4	0.36
Sufentanil (ug)	64.5 ± 4.9	65.7 ± 6.5	0.27
Oxycodone (mg)	2.1 ± 2.8	3.1 ± 3.5	0.10
PCA	55 (100.0)	58 (100.0)	0.99
Dosage of muscle relaxants			
Rocuronium (mg)	104.6 ± 28.1	98.4 ± 28.4	0.25
Reversal of neuromuscular block			
Sugammadex sodium	55 (100.0)	58 (100.0)	0.99
Intraoperative transfusion			
Crystalloids	956.0 ± 299.1	932.1 ± 301.1	0.67
Colloid solution	248.0 ± 270.5	341.5 ± 253.0	0.06
Total fluids	1204.0 ± 357.4	1273.6 ± 344.7	0.29
Antiemetic	55 (100.0)	58 (100.0)	0.99
Intraoperative desaturation	2 (3.64)	1 (1.72)	0.61
Intraoperative hemodynamic instability	7 (12.7)	6 (10.3)	0.77

*Note:* CIVIA: combined intravenous and inhalation anesthesia.

Abbreviations: JJB, jejunal–jejunal bypass; PCA, patient-controlled analgesia; SG, sleeve gastrectomy.

**Table 3:** Postoperative outcomes.

Variables	Low FiO_2_ group (*n* = 55)	High FiO_2_ group (*n* = 58)	Relative risk (95% CI)	*p* value
Primary outcomes				
PPCs	30 (55.5)	41 (70.7)	0.77 (0.57–1.03)	0.08
PPCs severity				
Grade 0	25 (45.5)	17 (29.3)	1.55 (0.96–2.56)	0.08
Grade 1	9 (16.4)	13 (22.4)	0.73 (0.34–1.54)	0.48
Grade 2	21 (38.1)	26 (44.8)	0.85 (0.54–1.32)	0.57
Grade 3	0 (0.0)	2 (3.4)	0.00 (0.00–1.98)	0.50
Grade 4	0 (0.0)	0 (0.0)	NA	0.99
Grade 5	0 (0.0)	0 (0.0)	NA	0.99
PPCs severity score	0.9 ± 0.9	1.2 ± 0.9	NA	0.08
PPCs severity grade ≥ 3	0 (0.0)	2 (3.4)	0.00 (0.00–1.98)	0.50
Secondary outcomes				
Length of hospital stay (day)	2.1 ± 0.3	2.1 ± 0.3	NA	0.99
In-hospital mortality	0 (0.0)	0 (0.0)	NA	0.99
30-day mortality	0 (0.0)	0 (0.0)	NA	0.99
Other outcomes				
PONV	24 (43.6)	16 (27.6)	1.58 (0.96–2.66)	0.08
SSI	0 (0.0)	1 (1.7)	0.00 (0.00–3.99)	0.99
Oxygen supplementation in PACU (hour)	1.0 ± 0.4	1.1 ± 0.5	NA	0.24

Abbreviations: CI, confidence interval; PONV, postoperative nausea and vomiting; PPCs, postoperative pulmonary complications; SSI, surgical site infection.

## Data Availability

The datasets are available from the corresponding author on reasonable request.

## Supporting Information

Supporting Information 1 CONSORT 2025 checklist.

Supporting Information 2 Table S1. Characteristics of ventilation.
