# Supplementary material for: Low vs. High Inspiratory Oxygen Fraction During Mechanical Ventilation in Obese Patients: Impact on Postoperative Pulmonary Outcomes
Source: Anesthesiol Res Pract. 2025 Oct 6;2025:5336172. doi: 10.1155/anrp/5336172 (PMC12517982; doi:10.1155/anrp/5336172)
Supplement: Supporting Information 2 — Table S1. Characteristics of ventilation. [file 5336172.f2.docx]

Table S1. Characteristics of ventilation.

|  | Low FiO2 group (n = 55) | High FiO2 group (n = 58) | *P* value |
| --- | --- | --- | --- |
| **Tidal volume (mL/kg)** |  |  |  |
| After intubation | 7.0 ± 0.1 | 7.0 ± 0.0 | 0.99 |
| 1f after surgery | 7.0 ± 0.1 | 7.0 ± 0.1 | 0.99 |
| End of surgery | 7.0 ± 0.1 | 7.0 ± 0.0 | 0.99 |
| **PEEP level (cm H2O)** |  |  |  |
| After intubation | 8.0 ± 0.0 | 8.0 ± 0.0 | NA |
| 1f after surgery | 8.0 ± 0.0 | 8.0 ± 0.0 | NA |
| End of surgery | 8.0 ± 0.0 | 8.0 ± 0.0 | NA |
| **P_peak_ (cmH2O)** |  |  |  |
| After intubation | 20.8 ± 3.4 | 20.9 ± 3.6 | 0.87 |
| 1f after surgery | 26.5 ± 2.8 | 26.4 ± 2.9 | 0.85 |
| End of surgery | 21.5 ± 3.1 | 21.6 ± 3.5 | 0.87 |
| **P_plat_ (cmH2O)** |  |  |  |
| After intubation | 20.3 ± 3.5 | 19.7 ± 3.3 | 0.35 |
| 1f after surgery | 25.6 ± 2.9 | 25.4 ± 2.9 | 0.71 |
| End of surgery | 20.5 ± 2.8 | 19.6 ± 3.4 | 0.12 |
| **Driving pressure (cmH2O)** |  |  |  |
| After intubation | 12.3 ± 3.5 | 11.8 ± 3.3 | 0.43 |
| 1f after surgery | 17.5 ± 2.9 | 17.4 ± 3.0 | 0.85 |
| End of surgery | 12.5 ± 2.8 | 11.7 ± 3.2 | 0.16 |
| **C_dyn_ (ml/cmH2O)** |  |  |  |
| After intubation | 32.2 ± 6.0 | 33.5 ± 7.5 | 0.31 |
| 1f after surgery | 22.9 ± 3.3 | 23.9 ± 4.3 | 0.17 |
| End of surgery | 30.9 ± 5.5 | 31.5 ± 6.8 | 0.60 |
| **Oxygenation index** |  |  |  |
| Baseline | 406.8 ± 57.0 | 414.0 ± 45.9 | 0.46 |
| 1h after surgery | 292.2 ± 75.0 | 304.1 ± 79.6 | 0.41 |
| After extubation | 341.8 ± 67.3 | 331.9 ± 59.1 | 0.40 |
| **PaCO2** |  |  |  |
| Baseline | 40.2 ± 7.5 | 38.5 ± 3.3 | 0.11 |
| 1h after surgery | 49.9 ± 7.8 | 48.3 ± 5.0 | 0.19 |
| After extubation | 48.3 ± 8.5 | 46.4 ± 3.8 | 0.12 |

PEEP, positive end expiratory pressure; P_peak_, peak airway pressure; P_plat_, plateau pressure; C_dyn_, dynamic compliance; PaO2, arterial partial pressure of oxygen; PaCO2, arterial partial pressure of carbon dioxide.
